# Supplementary material for: Screening and Verification of Photosynthesis and Chloroplast-Related Genes in Mulberry by Comparative RNA-Seq and Virus-Induced Gene Silencing
Source: Int J Mol Sci. 2022 Aug 3;23(15):8620. doi: 10.3390/ijms23158620 (PMC9368790; doi:10.3390/ijms23158620)
Supplement: Supplementary file 1 [file ijms-23-08620-s001.zip › ijms-1744990-supp.pdf]

Figures S1-S7

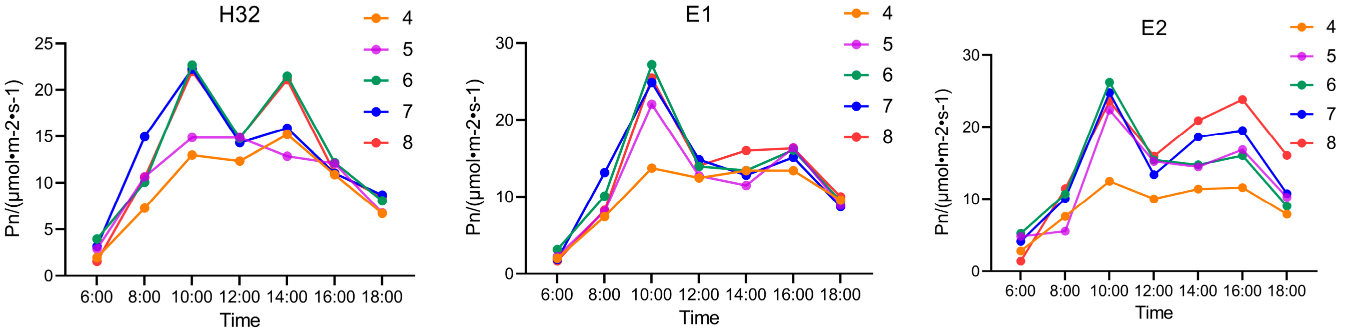

Figure S1 Dynamic changes of net photosynthetic rate (Pn) of mulberry leaves at different leaf positions. 4,5,6,7,8 in the legend represent leaf positions 4, leaf positions 5, leaf positions 6, leaf positions 7, leaf positions 8 , respectively.

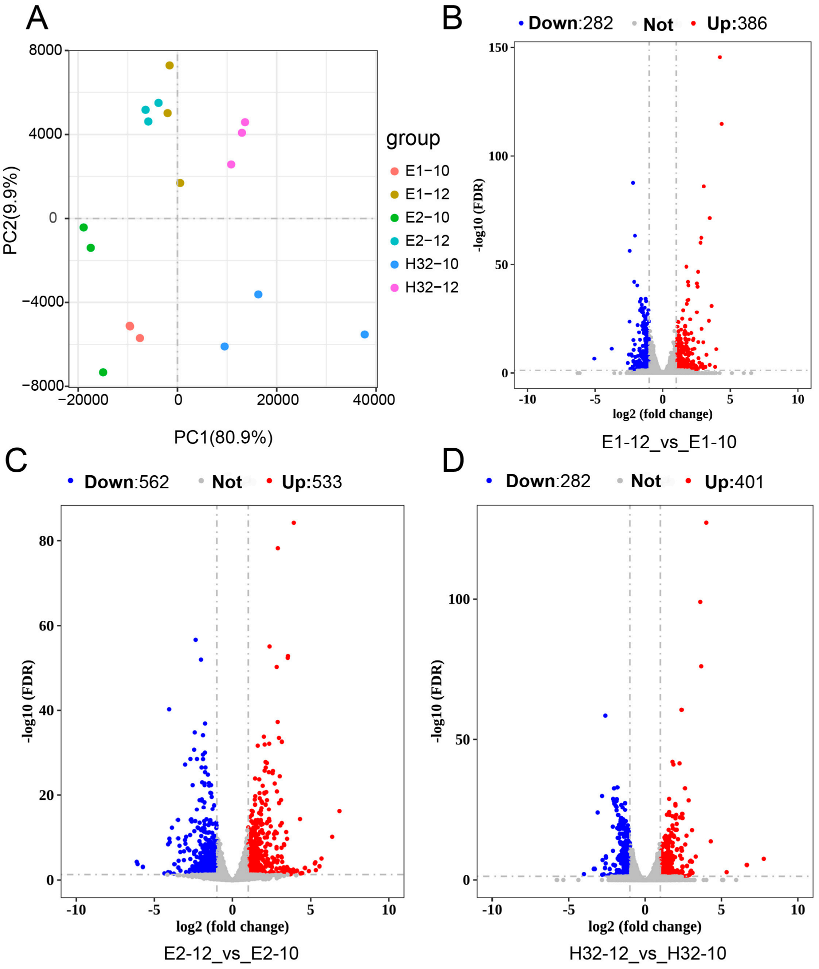

Figure S2. PCA analysis and Volcano map from DEGs. **(A)** PCA analysis; X-axis represents PC1 level, and Y-axis represents PC2 level; **(B)** Volcano map of DEGs in E1-12_vs_E1-10. **(C)** Volcano map of DEGs in E2-12_vs_E2-10 **(D)** Volcano map of DEGs in H32-12_vs_H32-10; The red, blue and gray dots represent up-regulated DEGs (Up), down-regulated DEGs (Down), and unaltered genes (Not), respectively.

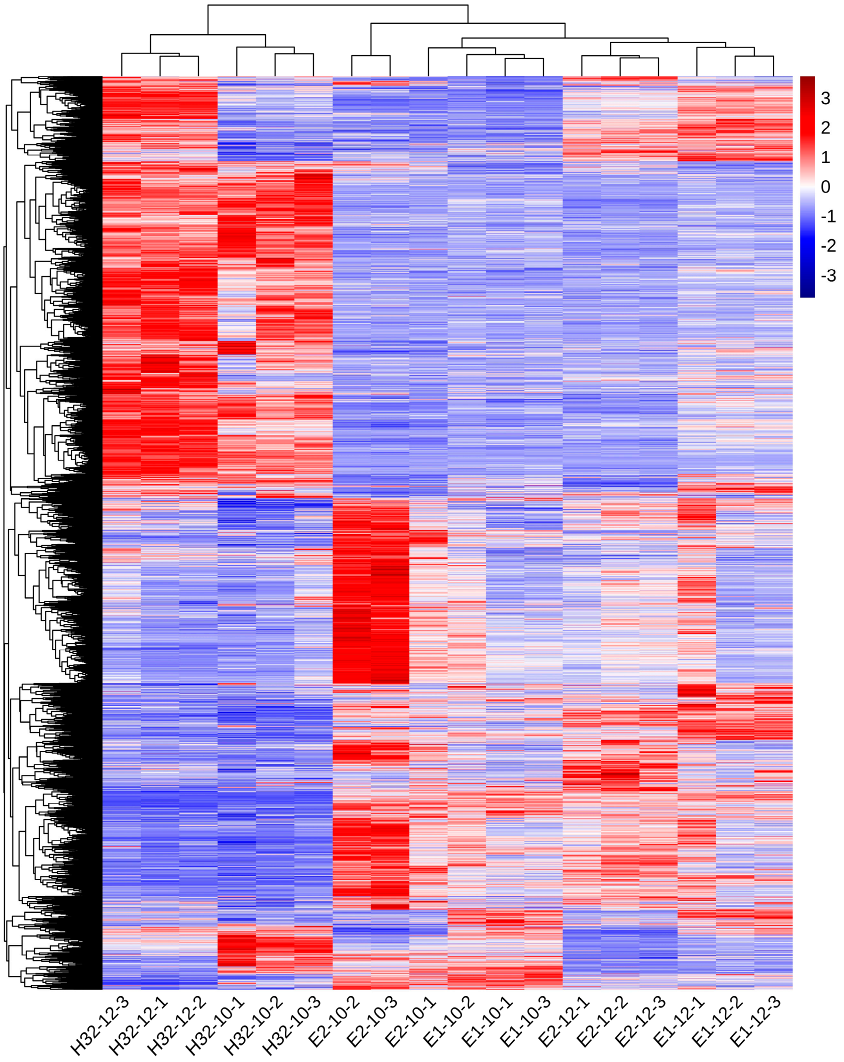

Figure S3. The heatmap of the DEGs. The abscissa is the materials and period in 18 samples; Red represents upregulation genes and blue represents downregulation genes; The color shading represents the degree of up- or down-regulation.

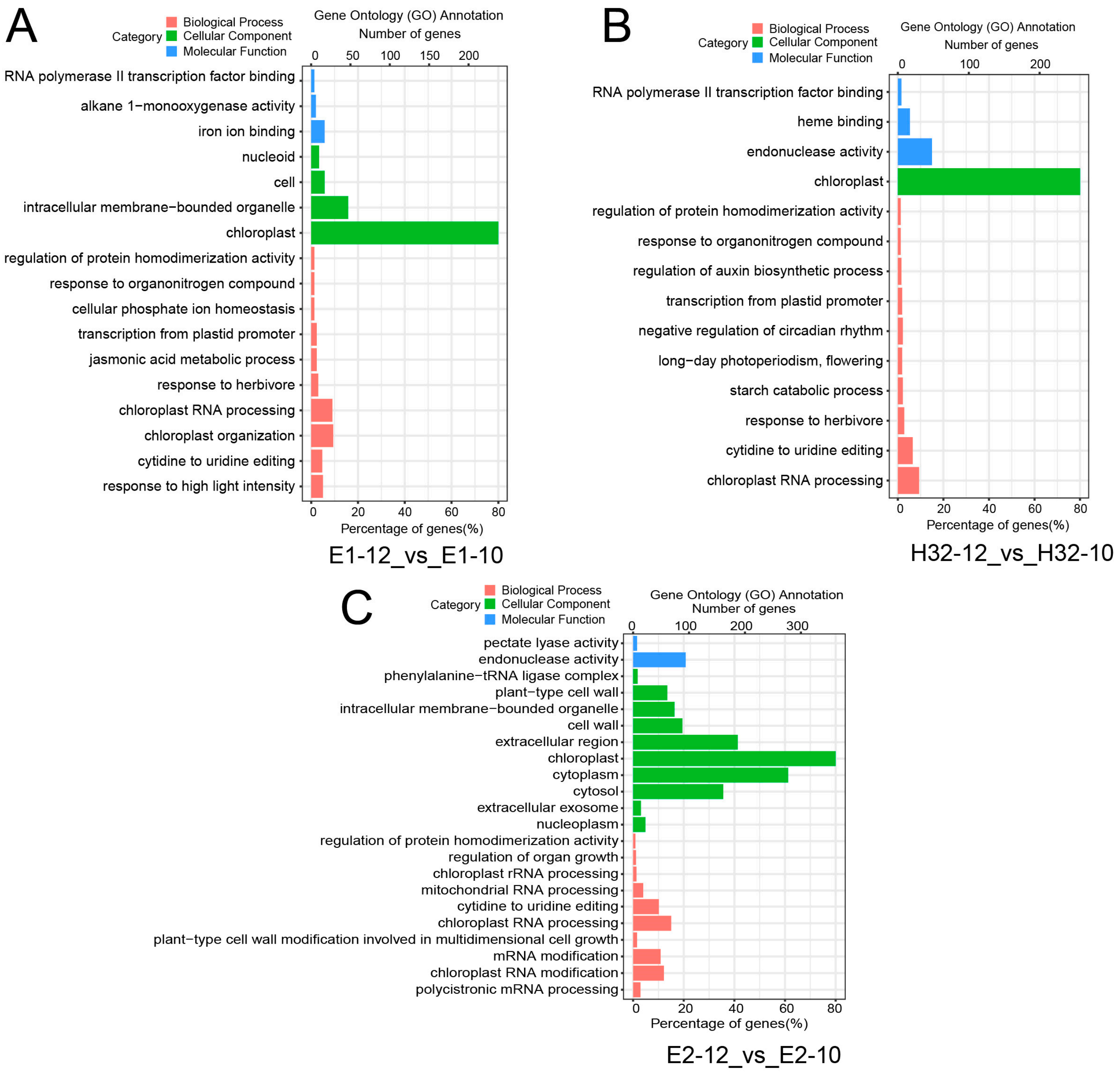

Figure S4. Gene ontology (GO) analysis of DEGs. The Y-axis represents the GO classification, the bottom and top of the X-axis represent the percentage and the number of genes, respectively.

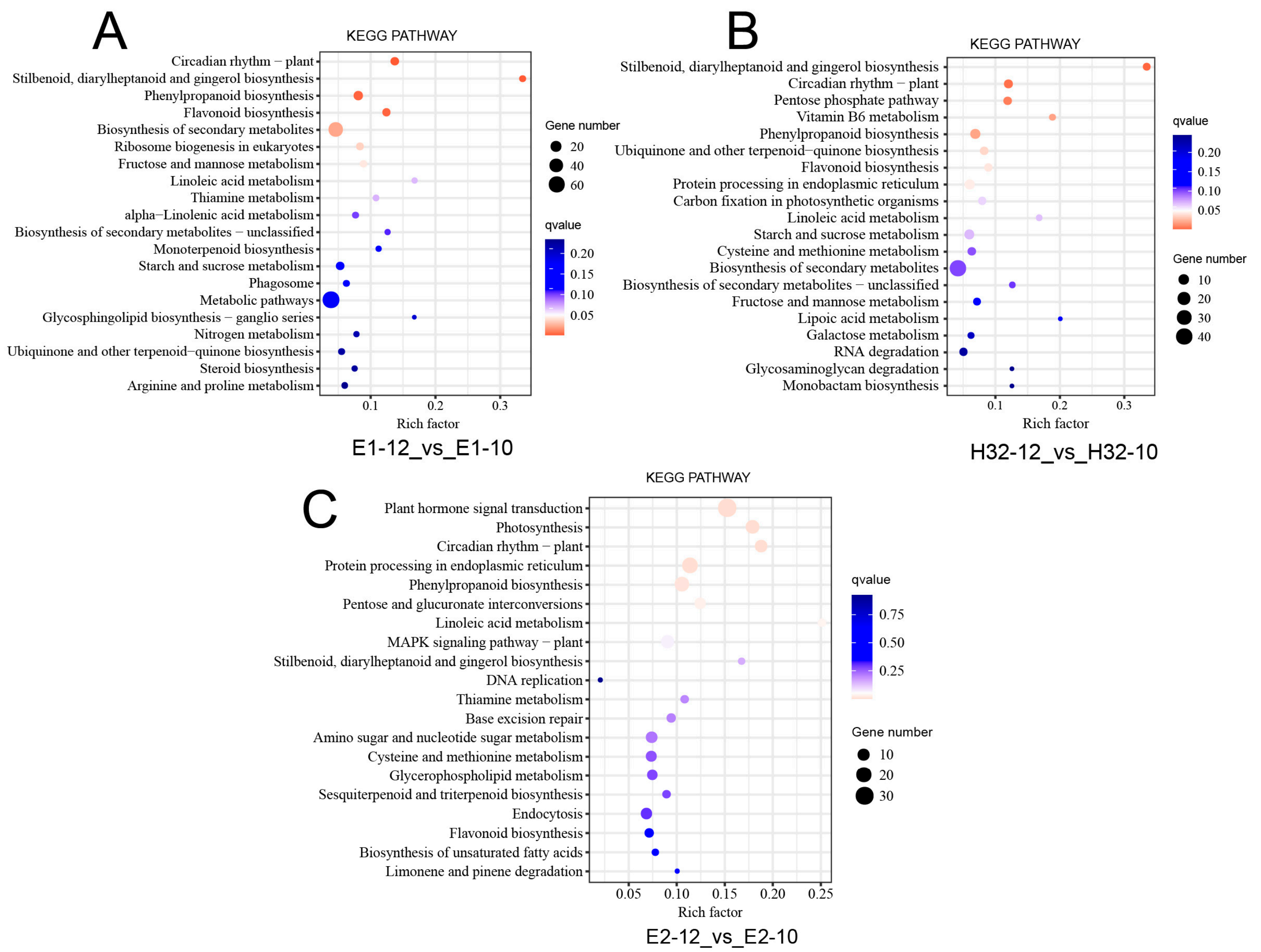

Figure S5. Kyoto Encyclopedia of Genes and Genomes pathway (KEGG) analysis of DEGs. Y-axis represents the pathway name, and X-axis represents the enrichment factor.

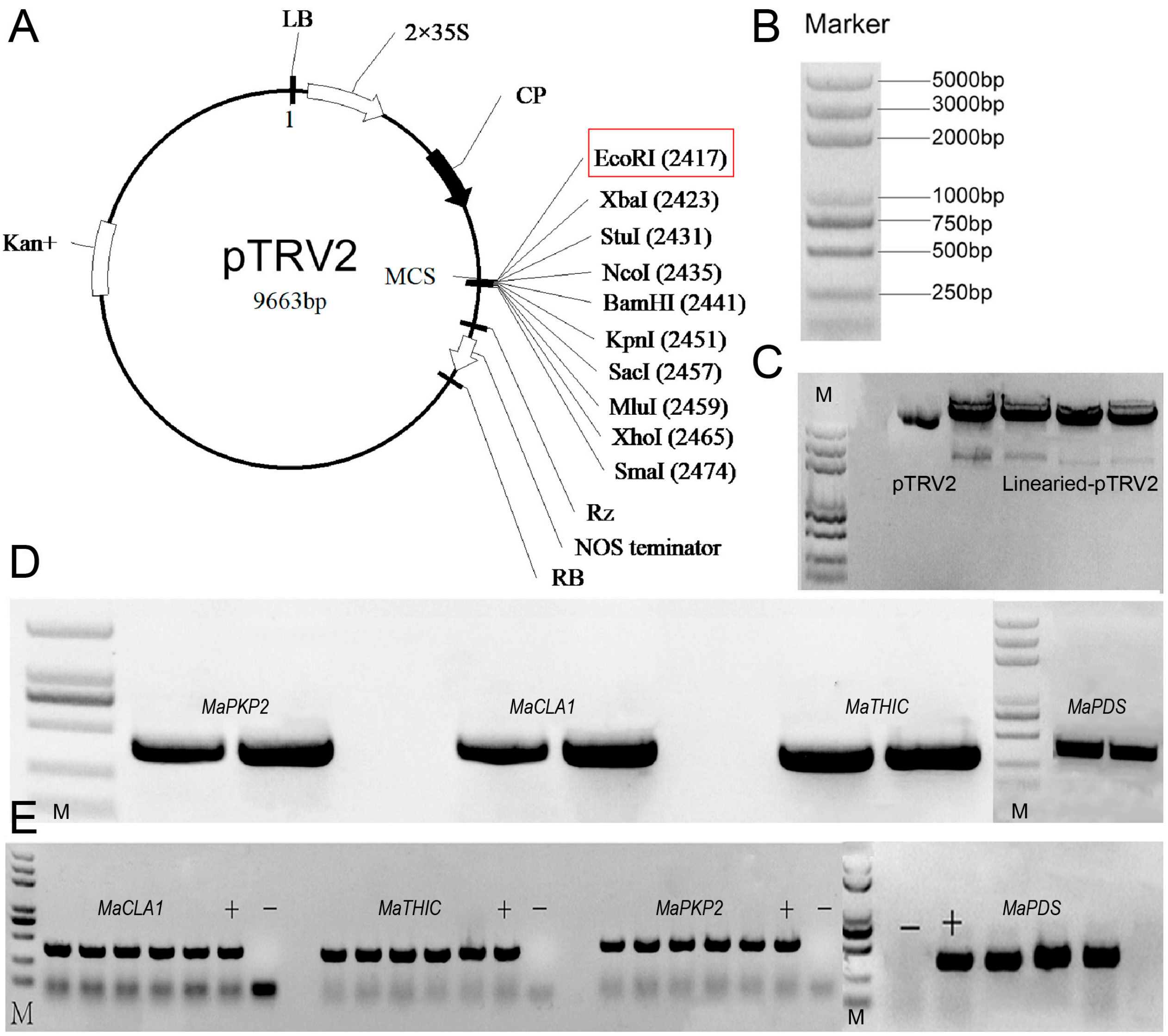

Figure S6. Construction of VIGS vectors for *MaCLA1*, *MaTHIC* and *MaPKP2*. **(A)** Vector map of pTRV2; **(B)** the size of maker; **(C)** digestion of pTRV2 vector with *EcoR*I enzyme; **(D)** The amplification of a fragment from *MaCLA1*, *MaTHIC* and *MaPKP2*. **(E)** Escherichia coli positive colony detection, + means positive control, - means negative control (water).

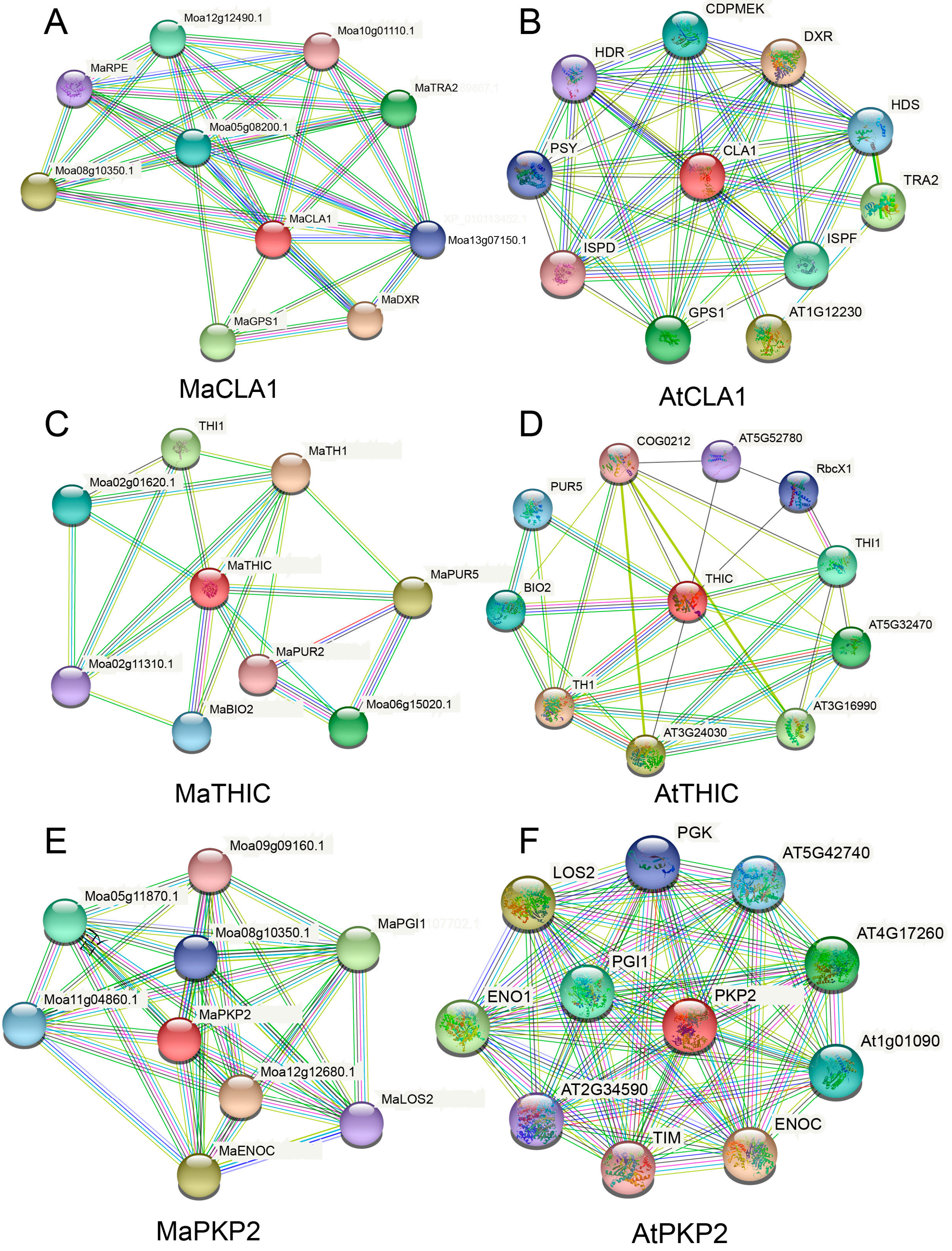

Figure S7. Predicted protein-protein interaction network (PPI) for MaCLA1, MaTHIC and MaPKP2 in Mubbery and *Arabidopsis*. STRING was used to predict it.

Tables S1-S9

Table S1. The statistics of mulberry transcriptome sequencing da data.

Sample	Raw data							Clean data						
	total_reads	total_bases	Q20_bases	Q30_bases	Q20_rate	Q30_rate	GC	total_reads	total_bases	Q20_bases	Q30_bases	Q20_rate	Q30_rate	GC
E1-10-1	50938182	7623856928	7433095199	7182889741	97.5	94.216	46.9133	49904388	7467213614	7324147857	7100995909	98.0841	95.0957	46.9698
E1-10-2	57502410	8604626164	8398232268	8123707404	97.6	94.4109	46.8577	56420992	8440697006	8284017696	8037613288	98.1438	95.2245	46.9188
E1-10-3	55283710	8274055786	8077414775	7816222486	97.62	94.4666	47.0436	54262738	8119051866	7969450644	7734832293	98.1574	95.2677	47.1037
E1-12-1	60485732	9051224190	8834171133	8546811229	97.6	94.4271	46.7147	59327236	8875399328	8711544030	8454080134	98.1538	95.253	46.7806
E1-12-2	51922802	7769419220	7582112745	7335476919	97.59	94.4147	46.7984	50923264	7617712288	7476225005	7255238529	98.1427	95.2417	46.8754
E1-12-3	57607004	8624107746	8406031377	8121483966	97.47	94.1719	46.7607	56418276	8444004528	8280572645	8026792175	98.0645	95.0591	46.8387
E2-10-1	56721894	8484822514	8277594718	8002981544	97.56	94.3211	47.0268	55613350	8316792792	8160631233	7915050952	98.1223	95.1695	47.0826
E2-10-2	48599692	7267499174	7101091221	6877721692	97.71	94.6367	46.8455	47755332	7139175358	7011519498	6809979439	98.2119	95.3889	46.9111
E2-10-3	58655292	8775608922	8571064743	8297233387	97.67	94.5488	46.6933	57610636	8616931722	8460574081	8214274814	98.1855	95.3271	46.7431
E2-12-1	54097150	8088905244	7891636882	7629527127	97.56	94.3209	47.3196	53042616	7929202016	7780372317	7545849672	98.123	95.1653	47.3795
E2-12-2	64950762	9712036986	9485938581	9181467440	97.67	94.537	47.2888	63794488	9536536538	9363700414	9089602536	98.1876	95.3135	47.3442
E2-12-3	52903402	7909894670	7718693769	7463322877	97.58	94.3543	47.348	51907882	7758882574	7613636304	7384486873	98.128	95.1746	47.4009
H32-10-1	46272192	6912557638	6768852364	6575400789	97.92	95.1225	46.8376	45519424	6797741860	6687723356	6512474516	98.3815	95.8035	46.9381
H32-10-2	54749408	8191543948	7999918406	7746128444	97.66	94.5625	45.9232	53754266	8040293906	7893980197	7665434545	98.1802	95.3377	45.9991
H32-10-3	52825708	7900236418	7723598804	7490201474	97.76	94.8098	46.087	51907008	7760166618	7625083369	7414367861	98.2593	95.5439	46.1783
H32-12-1	54295330	8123228686	7935248288	7688129011	97.69	94.6438	45.672	53312124	7973647018	7830248597	7607752670	98.2016	95.4112	45.7527
H32-12-2	54480328	8157104640	7951559824	7689080475	97.48	94.2624	45.5465	53322894	7981602870	7828829698	7595552318	98.0859	95.1632	45.6377
H32-12-3	58654694	8779792552	8566172042	8289745211	97.57	94.4185	45.8474	57476448	8600905094	8440920195	8194204318	98.1399	95.2714	45.9369

Table S2. The statistics of comparison between transcriptome data and *Morus notabilis* reference genome.

Sample	Total reads	Mapped reads	Unique-mapped reads	Multi-mapped reads	Total alignments(%)	Unique alignments(%)	Multi alignments(%)
E1-10-1	49904388	36294462	35109688	1184774	72.73	70.35	2.37
E1-10-2	56420992	40784486	39451935	1332551	72.29	69.92	2.36
E1-10-3	54262738	39282248	37985871	1296377	72.39	70	2.39
E1-12-1	59327236	42001780	40538273	1463507	70.8	68.33	2.47
E1-12-2	50923264	36381522	35097774	1283748	71.44	68.92	2.52
E1-12-3	56418276	39211083	37846516	1364567	69.5	67.08	2.42
E2-10-1	55613350	40461173	39134249	1326924	72.75	70.37	2.39
E2-10-2	47755332	34544951	33425737	1119214	72.34	69.99	2.34
E2-10-3	57610636	42180478	40779212	1401266	73.22	70.78	2.43
E2-12-1	53042616	37078826	35812395	1266431	69.9	67.52	2.39
E2-12-2	63794488	45421591	43848158	1573433	71.2	68.73	2.47
E2-12-3	51907882	37061768	35787516	1274252	71.4	68.94	2.45
H32-10-1	45519424	32319098	31223669	1095429	71	68.59	2.41
H32-10-2	53754266	37774487	36560293	1214194	70.27	68.01	2.26
H32-10-3	51907008	36035026	34858345	1176681	69.42	67.16	2.27
H32-12-1	53312124	36697868	35443635	1254233	68.84	66.48	2.35
H32-12-2	53322894	36822602	35556643	1265959	69.06	66.68	2.37
H32-12-3	57476448	39764262	38382832	1381430	69.18	66.78	2.4

Table S3. The number of GO terms with significant enrichment of DEGs.

Compare	Molecular Function	Biological Process	Cellular Component	Total
E1-10_vs_H32-10	85	137	47	269
E1-12_vs_E1-10	57	148	34	239
E1-12_vs_H32-12	80	140	49	269
E2-10_vs_E1-10	71	129	38	238
E2-10_vs_H32-10	83	146	42	271
E2-12_vs_E1-12	64	118	24	206
E2-12_vs_E2-10	74	173	39	286
E2-12_vs_H32-12	76	135	36	247
H32-12_vs_H32-10	56	163	26	245
Total	646/310	1289/624	335/126	2270/1060

Note: The table shows the number of GO terms significantly enriched by all differential genes, with the number before "/" is significantly enriched to the total number of GO terms in the three groups without duplication, and the number after "/" is the total number of GO terms enriched by the different genes into the three groups after weight reduction

Table S4. Expression of photosynthetic and chloroplast genes in 7 groups.

Number	gene_ID	At_gene	identify%)	gene_name	E1-10_vs_H32-10	E1-12_vs_H32-12	E2-10_vs_H32-10	E2-12_vs_H32-12	E1-12_vs_E1-10	E2-12_vs_E2-10	H32-12_vs_H32-10
1	Moa01g06130.1	AT3G01440.1	60	PnsL3	Down	Down	Down	Down	NA	NA	NA
2	Moa08g02040.1	AT1G16540.1	65.043	ABA3	NA	Down	NA	Down	NA	NA	NA
3	Moa12g06150.1	AT1G60600.2	68.73	ABC4	NA	NA	NA	NA	NA	NA	Up
4	Moa13g02830.1	AT4G37640.1	82.857	ACA2	NA	NA	Down	Down	NA	NA	NA
5	Moa06g11500.1	AT2G28800.1	79.73	ALB3	NA	NA	Down	NA	NA	NA	NA
6	Moa06g05350.1	AT1G08980.1	66.029	AMI1	NA	NA	Down	NA	NA	NA	NA
7	Moa09g05340.1	AT4G00370.1	81.25	ANTR2	NA	NA	Down	NA	Down	NA	Down
8	Moa10g07110.1	AT5G38660.1	77.434	APE1	NA	NA	Down	NA	NA	NA	NA
9	Moa04g05100.1	AT5G46110.1	81.055	APE2	NA	NA	Down	NA	NA	NA	NA
10	Moa07g03660.1	AT3G63410.1	76.369	APG1	NA	NA	NA	NA	NA	NA	Down
11	Moa14g07080.1	AT2G01110.1	61.511	APG2	NA	NA	Down	NA	NA	NA	NA
12	Moa05g17710.1	AT4G32320.1	60.577	APX6	NA	NA	Down	Down	NA	NA	NA
13	Moa06g09450.1	AT3G47340.1	86.583	ASN1	Down	Down	Down	Down	NA	NA	NA
14	pgp057	ATCG00480.1	94.98	atpB	NA	NA	NA	Up	NA	Up	NA
15	pgp078	ATCG00130.1	85.326	atpF	NA	NA	NA	Up	NA	NA	NA
16	pgp077	ATCG00140.1	98.765	atpH	NA	NA	NA	Up	NA	NA	NA
17	Moa02g12160.1	AT3G23920.1	67.327	BAM1	NA	NA	NA	NA	Down	NA	Down
18	Moa05g15110.1	AT1G10070.2	73.156	BCAT-2	NA	Down	NA	NA	NA	NA	NA
19	Moa14g02910.1	AT1G23400.1	61.67	CAF2	NA	NA	Down	NA	NA	Up	NA
20	Moa05g05680.1	AT4G32810.1	62.937	CCD8	NA	NA	NA	Down	NA	NA	NA
21	pgp052	ATCG00530.1	74.892	cemA	NA	NA	NA	NA	NA	Up	NA
22	Moa04g00510.1	AT5G44650.1	70.225	CEST	Down	NA	Down	NA	NA	NA	NA
23	Moa02g05990.1	AT3G23070.1	71.175	CFM3A	NA	NA	Down	NA	NA	NA	NA
24	Moa12g01070.1	AT2G37770.2	68.932	ChlAKR	Down	NA	NA	NA	NA	NA	NA
25	Moa14g07620.1	AT3G25690.2	66.035	CHUP1	NA	NA	NA	NA	Down	NA	Down
26	Moa06g15270.1	AT1G08640.1	65.217	CJD1	NA	NA	NA	NA	Up	NA	NA
27	**Moa02g03470.1**	**AT4G15560.1**	**87.593**	**CLA1**	**NA**	**NA**	**NA**	**Up**	**Up**	**NA**	**NA**
28	Moa03g09030.1	AT3G52380.1	68.807	CP33	NA	NA	Down	NA	NA	Up	NA
29	Moa04g01430.1	AT5G18820.1	75.543	Cpn60alpha2	NA	NA	NA	NA	Up	NA	NA
30	Moa14g06540.1	AT1G26230.1	75.741	Cpn60beta4	NA	NA	NA	NA	NA	NA	Up
31	Moa06g10650.1	AT3G46790.1	70.076	CRR2	NA	NA	NA	NA	NA	Up	NA
32	Moa09g07210.1	AT2G45350.1	64.421	CRR4	NA	NA	Down	NA	Up	Up	Up
33	Moa06g00270.1	AT2G28190.1	73.094	CSD2	NA	Up	NA	NA	NA	NA	NA
34	Moa12g07270.1	AT4G17090.1	73.042	CT-BMY	NA	NA	NA	Down	NA	NA	Up
35	Moa07g02630.1	AT1G63900.1	76.093	DAL1	Down	Down	Down	Down	NA	NA	NA
36	Moa10g02670.1	AT3G27925.1	88.493	DEG1	NA	NA	Down	NA	NA	NA	NA
37	Moa07g12550.1	AT4G34020.1	66.133	DJ1C	NA	NA	NA	NA	NA	Up	NA
38	Moa03g04110.1	AT2G40840.1	73.769	DPE2	Down	NA	Down	Down	Up	Up	Up

39	Moa02g13070.1	AT1G15510.1	65.862	ECB2	NA	NA	NA	NA	NA	Up	NA
40	Moa07g13520.1	AT4G34200.1	84.237	EDA9	NA	NA	Up	NA	NA	NA	NA
41	Moa02g05330.1	AT3G22840.1	68.675	ELIP1	NA	NA	NA	NA	Down	Down	Down
42	Moa07g15800.1	AT3G18110.1	75.309	EMB1270	NA	NA	Down	Down	Up	Up	Up
43	Moa14g01210.1	AT1G67440.1	69.637	emb1688	Down	Down	NA	Down	NA	NA	NA
44	Moa11g02010.1	AT3G49240.1	62.937	emb1796	NA	NA	NA	NA	Up	Up	NA
45	Moa06g17130.1	AT3G12080.1	76.84	emb2738	NA	Down	NA	Down	NA	NA	NA
46	Moa08g02920.1	AT5G63420.1	73.035	emb2746	NA	NA	Down	NA	NA	Up	NA
47	Moa04g11340.1	AT3G15850.1	75.932	FAD5	NA	NA	NA	NA	NA	NA	Up
48	Moa05g01050.1	AT1G43670.1	88.657	FBP	NA	NA	NA	NA	NA	NA	Down
49	Moa11g06970.1	AT3G11050.1	73.256	FER2	NA	NA	NA	NA	Down	Down	Down
50	Moa06g16160.1	AT1G79790.1	65.025	FHY1	Down	NA	Down	Down	NA	NA	NA
51	Moa12g01710.1	AT3G54090.1	70.41	FLN1	NA	NA	NA	NA	Up	Up	Up
52	Moa14g05970.1	AT1G69200.1	63.729	FLN2	NA	NA	NA	NA	Up	NA	NA
53	Moa11g00290.1	AT1G76320.1	65.462	FRS4	NA	Down	NA	NA	NA	NA	NA
54	Moa06g10030.1	AT5G58870.1	72.979	ftsh9	NA	NA	Down	NA	NA	NA	NA
55	Moa07g01390.1	AT2G47510.2	87.652	FUM1	NA	NA	Up	NA	NA	NA	NA
56	Moa07g00770.1	AT2G31400.1	68.187	GUN1	NA	NA	NA	NA	Up	Up	Up
57	Moa14g11510.1	AT1G79000.1	61.323	HAC1	NA	NA	NA	NA	Down	NA	Down
58	Moa09g13170.1	AT3G24430.1	88.839	HCF101	Down	Down	Down	Down	NA	NA	NA
59	Moa07g11460.1	AT4G34350.1	78.326	HDR	NA	NA	Down	NA	NA	NA	NA
60	Moa02g12570.1	AT5G08280.1	84.956	HEMC	Down	NA	Down	Down	Up	NA	NA
61	Moa09g05780.1	AT4G00630.1	72.426	KEA2	NA	NA	Up	Up	NA	NA	NA
62	Moa04g07390.1	AT2G34430.1	89.098	LHB1B1	NA	NA	NA	Up	NA	NA	NA
63	Moa06g17510.1	AT3G45140.1	60.979	LOX2	NA	Up	NA	NA	Up	Up	Up
64	Moa14g05410.1	AT1G69390.1	62.832	MINE1	NA	NA	NA	Up	NA	NA	NA
65	Moa01g06770.1	AT1G11430.1	80.214	MORF9	Down	Down	Down	Down	NA	NA	NA
66	Moa07g04750.1	AT4G28590.1	60.169	MRL7	Down	Down	Down	Down	NA	NA	NA
67	Moa02g05740.1	AT3G23020.1	60.093	PPR30	NA	NA	NA	NA	NA	Up	NA
68	Moa07g02640.1	AT1G54790.1	60.317	NA	Up	NA	Up	Up	Down	NA	NA
69	Moa02g05110.1	AT3G22690.2	60.377	YS1	NA	NA	NA	NA	NA	Up	NA
70	Moa09g08310.1	AT1G01320.2	60.808	REC1	NA	Down	Down	NA	NA	NA	NA
71	Moa01g15910.1	AT5G20180.2	62.136	NA	NA	Down	Down	Down	NA	NA	NA
72	Moa14g06450.1	AT1G68930.1	62.719	NA	NA	NA	NA	NA	NA	Up	NA
73	Moa07g14230.1	AT2G21340.1	63.225	NA	Down	Down	Down	Down	NA	NA	NA
74	Moa10g06210.1	AT5G15700.1	63.725	NA	NA	NA	NA	NA	NA	Up	NA
75	Moa12g11870.1	AT4G22840.1	64.891	NA	NA	NA	Down	NA	NA	NA	NA
76	Moa03g08910.1	AT4G28360.1	64.964	NA	Down	Down	Down	Down	NA	NA	NA
77	Moa14g07560.1	AT4G13180.1	65.37	NA	NA	NA	Up	NA	NA	NA	NA
78	Moa03g12740.1	AT2G37220.1	68.376	NA	NA	NA	Down	NA	Up	Up	NA
79	Moa08g11630.1	AT5G53490.1	69.194	NA	Down	Down	Down	Down	NA	Up	NA

80	Moa08g04620.1	AT5G63060.1	69.397	NA	NA	NA	NA	Down	NA	NA	NA
81	Moa12g06840.1	AT5G46580.1	69.48	NA	NA	NA	NA	NA	Up	Up	Up
82	Moa13g03000.1	AT5G67385.1	70.323	NA	NA	NA	Up	NA	NA	NA	NA
83	Moa02g09190.1	AT3G23700.1	71.141	NA	NA	NA	Down	Down	NA	NA	NA
84	Moa08g09220.1	AT1G68660.1	71.25	NA	NA	NA	Down	NA	NA	NA	NA
85	Moa03g00620.1	AT3G03890.1	71.519	NA	NA	NA	NA	Up	NA	NA	NA
86	Moa12g01550.1	AT1G56500.1	76.3	NA	NA	NA	Down	NA	NA	NA	NA
87	Moa02g06360.1	AT4G14480.1	76.639	NA	Up	NA	NA	NA	NA	Up	NA
88	Moa10g13380.1	AT3G07700.2	76.912	NA	NA	NA	Down	NA	NA	NA	NA
89	Moa09g13910.1	AT3G60370.1	77.228	NA	NA	NA	Down	NA	NA	NA	NA
90	Moa03g14310.1	AT1G79600.1	77.647	NA	NA	NA	Down	NA	NA	NA	NA
91	Moa06g03210.1	AT5G19500.1	78.345	NA	NA	NA	Down	Down	NA	NA	NA
92	Moa07g00940.1	AT2G47390.1	81.408	NA	NA	NA	NA	NA	NA	NA	Up
93	Moa10g03540.1	AT5G39980.1	83.251	EMB3140	NA	NA	NA	NA	NA	Up	NA
94	Moa11g14660.1	AT1G16740.1	84.677	NA	Up	Up	Up	Up	NA	NA	NA
95	Moa06g18220.1	AT5G08650.1	85.312	NA	NA	NA	Down	NA	NA	NA	NA
96	Moa02g10410.1	AT4G19170.1	69.231	NCED4	NA	NA	Down	NA	NA	NA	NA
97	pgp009	ATCG01100.1	85.399	ndhA	NA	NA	NA	Up	NA	Up	NA
98	pgp014	ATCG01050.1	84.97	ndhD	NA	NA	NA	Up	NA	NA	NA
99	Moa08g12220.1	AT1G74880.1	67.683	NdhO	NA	NA	Down	NA	NA	NA	NA
100	Moa07g06690.1	AT5G59200.1	64.516	OTP80	NA	NA	NA	NA	NA	Up	NA
101	Moa06g09830.1	AT2G29760.1	60.377	OTP81	Up	NA	Up	Up	NA	Up	Up
102	Moa01g09900.1	AT1G08070.1	66.584	OTP82	NA	NA	NA	NA	NA	Up	Up
103	Moa04g00100.1	AT3G57430.1	65.682	OTP84	Up	NA	NA	NA	NA	Up	NA
104	Moa14g04910.1	AT2G02980.1	67.295	OTP85	Up	Up	Up	Up	Up	Up	NA
105	Moa07g17460.1	AT3G13490.1	73.267	OVA5	NA	NA	NA	Up	NA	NA	NA
106	Moa07g02760.1	AT2G48120.1	67.29	PAC	NA	NA	NA	NA	NA	NA	Up
107	Moa06g00960.1	ATCG00540.1	76.19	petA	Down	Down	Down	Down	NA	NA	NA
108	pgp033	ATCG00730.1	98.758	petD	NA	NA	NA	Up	NA	Up	NA
109	Moa04g11330.1	AT3G15840.1	64.643	PIFI	NA	NA	NA	NA	NA	Up	NA
110	**Moa11g02720.1**	**AT5G52920.1**	**84.974**	**PKP2**	**NA**	**NA**	**Up**	**NA**	**NA**	**NA**	**NA**
111	Moa02g03620.1	AT4G15530.4	81.953	PPDK	NA	Down	Down	NA	NA	NA	NA
112	pgp064	ATCG00350.1	97.867	psaA	NA	NA	NA	Up	NA	Up	NA
113	pgp065	ATCG00340.1	97.684	psaB	NA	NA	NA	Up	NA	Up	NA
114	pgp038	ATCG00680.1	97.441	psbB	NA	NA	NA	Up	NA	NA	NA
115	pgp068	ATCG00280.1	97.674	psbC	NA	NA	NA	Up	NA	Up	NA
116	pgp080	ATCG00080.1	100	psbI	NA	NA	NA	NA	NA	Up	NA
118	Moa08g04120.1	AT5G50210.1	71.92	QS	NA	NA	Down	NA	NA	NA	Down
119	pgp056	ATCG00490.1	94.737	rbcL	NA	NA	NA	Up	NA	Up	NA
120	Moa04g17560.1	AT4G04330.1	74.242	RbcX1	NA	NA	NA	NA	Up	Up	Up
121	Moa05g02340.1	AT4G09730.1	67.427	RH39	NA	NA	NA	NA	Up	Up	Up

122	Moa03g14180.1	AT3G03710.1	72.979	RIF10	NA	NA	Down	NA	NA	Up	NA
123	Moa07g02560.1	AT2G48070.2	67.513	RPH1	Down	Down	Down	NA	NA	NA	NA
124	pgp074	ATCG00170.1	80.87	rpoC2	NA	NA	NA	Up	NA	Up	NA
125	pgp031	ATCG00750.1	92.754	rps11	NA	NA	NA	Up	NA	Up	NA
126	Moa03g00150.1	ATCG00330.1	92	rps14	NA	NA	NA	Up	NA	Up	NA
127	pgp062	ATCG00380.1	89.552	rps4	NA	NA	NA	Up	NA	Up	NA
128	Moa03g00240.1	ATCG00770.1	79.412	rps8	NA	NA	NA	Up	NA	NA	NA
129	Moa12g12370.1	AT1G62750.1	85.823	SCO1	NA	NA	NA	NA	Down	NA	Down
130	Moa05g19090.1	AT5G42390.1	73.684	SPP	Down	NA	Down	Down	NA	NA	NA
131	Moa05g09440.1	AT1G11870.2	72.5	SRS	NA	NA	Down	NA	NA	NA	NA
132	Moa07g08390.1	AT4G38470.1	72.727	STY46	NA	NA	NA	NA	Up	NA	NA
133	Moa07g03450.1	AT3G51895.1	76.888	SULTR3	NA	NA	NA	Up	Down	NA	Down
134	Moa03g06890.1	AT5G13650.1	88.368	SVR3	Down	Down	Down	Down	NA	NA	NA
135	**Moa06g10020.1**	**AT2G29630.3**	**86.574**	**THIC**	**NA**	**NA**	**NA**	**NA**	**Up**	**Up**	**Up**
136	Moa06g08770.1	AT1G06950.1	69.54	TIC110	Down	NA	NA	Down	NA	NA	NA
137	Moa07g15510.1	AT5G09420.1	64.026	TOC64-V	Down	NA	NA	NA	NA	NA	NA
138	Moa06g10910.1	AT3G46740.1	88.841	TOC75-III	NA	NA	NA	NA	Up	NA	Up
139	Moa02g08320.1	AT3G06730.1	78.621	TRXz	NA	NA	NA	NA	Up	Up	Up
140	Moa08g06300.1	AT1G74700.1	76.344	TRZ1	NA	NA	Down	NA	NA	NA	NA
141	Moa12g01220.1	AT3G53900.2	68.475	UPP	Down	NA	Down	NA	NA	NA	NA
142	pgp053	ATCG00520.1	87.5	ycf4	NA	NA	NA	Up	NA	NA	NA

Note: highlighted red are genes for VIGS experiments.

Table S5. Photosynthetic and chloroplast related genes in different materials.

Number	gene_ID	E1-10_vs_H32-10			E1-12_vs_H32-12			E2-10_vs_H32-10			E2-12_vs_H32-12		
		log2(FC)	FDR	Change	log2(FC)	FDR	Change	log2(FC)	FDR	Change	log2(FC)	FDR	Change
**1**	**Moa01g06130.1**	**-1.67596**	**0.001496**	**Down**	**-1.95581**	**1.37E-07**	**Down**	**-2.69617**	**1.04E-05**	**Down**	**-1.99415**	**1.73E-08**	**Down**
2	Moa08g02040.1	NA	NA	NA	-1.11973	0.000127	Down	NA	NA	NA	-1.01556	0.000204	Down
3	Moa13g02830.1	NA	NA	NA	NA	NA	NA	-1.34851	1.54E-09	Down	-1.04617	1.93E-17	Down
4	Moa06g11500.1	NA	NA	NA	NA	NA	NA	-1.22834	8.99E-07	Down	NA	NA	NA
5	Moa06g05350.1	NA	NA	NA	NA	NA	NA	-1.20676	1.19E-08	Down	NA	NA	NA
6	Moa09g05340.1	NA	NA	NA	NA	NA	NA	-1.03984	0.001595	Down	NA	NA	NA
7	Moa10g07110.1	NA	NA	NA	NA	NA	NA	-1.02825	0.000307	Down	NA	NA	NA
8	Moa04g05100.1	NA	NA	NA	NA	NA	NA	-1.15653	2.79E-05	Down	NA	NA	NA
9	Moa14g07080.1	NA	NA	NA	NA	NA	NA	-1.09552	1.41E-05	Down	NA	NA	NA
10	Moa05g17710.1	NA	NA	NA	NA	NA	NA	-1.19486	0.014725	Down	-1.08554	0.002751	Down
**11**	**Moa06g09450.1**	**-2.45269**	**3.67E-18**	**Down**	**-2.01514**	**1.45E-08**	**Down**	**-2.1391**	**9.72E-17**	**Down**	**-1.53096**	**1.24E-08**	**Down**
12	pgp057	NA	NA	NA	NA	NA	NA	NA	NA	NA	2.139343	0.004244	Up
13	pgp078	NA	NA	NA	NA	NA	NA	NA	NA	NA	3.731003	8.56E-05	Up
14	pgp077	NA	NA	NA	NA	NA	NA	NA	NA	NA	4.192261	0.004357	Up
15	Moa05g15110.1	NA	NA	NA	-1.68158	0.01252	Down	NA	NA	NA	NA	NA	NA
16	Moa14g02910.1	NA	NA	NA	NA	NA	NA	-1.76385	9.31E-13	Down	NA	NA	NA
17	Moa05g05680.1	NA	NA	NA	NA	NA	NA	NA	NA	NA	-4.32187	0.009113	Down
18	Moa04g00510.1	-1.17681	0.016901	Down	NA	NA	NA	-1.09008	0.004688	Down	NA	NA	NA
19	Moa02g05990.1	NA	NA	NA	NA	NA	NA	-1.01593	0.013299	Down	NA	NA	NA
20	Moa12g01070.1	-1.47077	6.03E-05	Down	NA	NA	NA	NA	NA	NA	NA	NA	NA
21	**Moa02g03470.1**	**NA**	**NA**	**NA**	**NA**	**NA**	**NA**	**NA**	**NA**	**NA**	**1.124965**	**0.004896**	**Up**
22	Moa03g09030.1	NA	NA	NA	NA	NA	NA	-1.40899	2.61E-09	Down	NA	NA	NA
23	Moa09g07210.1	NA	NA	NA	NA	NA	NA	-1.8591	9.90E-08	Down	NA	NA	NA
24	Moa06g00270.1	NA	NA	NA	1.465312	2.03E-11	Up	NA	NA	NA	NA	NA	NA
25	Moa12g07270.1	NA	NA	NA	NA	NA	NA	NA	NA	NA	-1.58506	2.99E-46	Down
**26**	**Moa07g02630.1**	**-1.2703**	**3.44E-09**	**Down**	**-1.2872**	**4.70E-11**	**Down**	**-1.11947**	**3.88E-06**	**Down**	**-1.06582**	**1.03E-11**	**Down**
27	Moa10g02670.1	NA	NA	NA	NA	NA	NA	-1.07642	6.24E-06	Down	NA	NA	NA
28	Moa03g04110.1	-1.12271	2.09E-07	Down	NA	NA	NA	-1.75866	2.00E-13	Down	-1.5425	8.04E-38	Down
29	Moa07g13520.1	NA	NA	NA	NA	NA	NA	1.757829	3.15E-10	Up	NA	NA	NA
30	Moa07g15800.1	NA	NA	NA	NA	NA	NA	-1.71582	3.32E-05	Down	-1.03146	1.92E-11	Down
31	Moa14g01210.1	-1.79849	0.008968	Down	-1.17868	0.021248	Down	NA	NA	NA	-1.0365	0.026413	Down
32	Moa06g17130.1	NA	NA	NA	-2.53153	0.01344	Down	NA	NA	NA	-1.82136	0.048084	Down
33	Moa08g02920.1	NA	NA	NA	NA	NA	NA	-1.51892	3.22E-05	Down	NA	NA	NA
34	Moa06g16160.1	-1.26397	0.023516	Down	NA	NA	NA	-2.25354	2.99E-05	Down	-1.7631	1.90E-05	Down
35	Moa11g00290.1	NA	NA	NA	-2.08327	0.046564	Down	NA	NA	NA	NA	NA	NA
36	Moa06g10030.1	NA	NA	NA	NA	NA	NA	-1.09014	2.51E-06	Down	NA	NA	NA
37	Moa07g01390.1	NA	NA	NA	NA	NA	NA	1.02955	0.000935	Up	NA	NA	NA
**38**	**Moa09g13170.1**	**-3.69003**	**3.29E-07**	**Down**	**-3.80759**	**2.31E-05**	**Down**	**-4.83416**	**3.73E-08**	**Down**	**-4.19035**	**9.25E-06**	**Down**
39	Moa07g11460.1	NA	NA	NA	NA	NA	NA	-1.19578	1.00E-07	Down	NA	NA	NA
40	Moa02g12570.1	-1.72869	7.38E-06	Down	NA	NA	NA	-1.45215	4.77E-05	Down	-1.14409	4.00E-05	Down
41	Moa09g05780.1	NA	NA	NA	NA	NA	NA	1.478686	0.033457	Up	1.210245	0.011671	Up
42	Moa04g07390.1	NA	NA	NA	NA	NA	NA	NA	NA	NA	1.050815	6.07E-61	Up
43	Moa06g17510.1	NA	NA	NA	1.402007	5.36E-29	Up	NA	NA	NA	NA	NA	NA
44	Moa14g05410.1	NA	NA	NA	NA	NA	NA	NA	NA	NA	1.10066	1.31E-10	Up
**45**	**Moa01g06770.1**	**-2.7146**	**1.66E-10**	**Down**	**-2.33067**	**3.75E-21**	**Down**	**-2.50908**	**4.75E-11**	**Down**	**-2.69216**	**2.13E-26**	**Down**
**46**	**Moa07g04750.1**	**-1.76744**	**1.16E-06**	**Down**	**-1.55377**	**4.63E-08**	**Down**	**-1.66072**	**6.90E-06**	**Down**	**-1.0364**	**4.35E-05**	**Down**
47	Moa07g02640.1	2.989051	0.003012	**Up**	NA	NA	NA	2.876973	0.005773	Up	2.485697	0.000122	Up
48	Moa09g08310.1	NA	NA	NA	-1.05489	8.40E-19	Down	-1.14495	0.000458	Down	NA	NA	NA
49	Moa01g15910.1	NA	NA	NA	-1.3238	0.000886	Down	-3.97518	2.49E-10	Down	-4.27805	1.80E-11	Down
**50**	**Moa07g14230.1**	**-1.66776**	**6.70E-06**	**Down**	**-2.24877**	**6.57E-11**	**Down**	**-1.54591**	**5.29E-05**	**Down**	**-1.22178**	**1.94E-05**	**Down**
51	Moa12g11870.1	NA	NA	NA	NA	NA	NA	-1.75161	0.005408	Down	NA	NA	NA
**52**	**Moa03g08910.1**	**-2.33653**	**7.95E-13**	**Down**	**-2.68097**	**3.13E-19**	**Down**	**-2.47205**	**1.15E-13**	**Down**	**-2.46629**	**1.27E-18**	**Down**
53	Moa14g07560.1	NA	NA	NA	NA	NA	NA	2.169342	0.028413	Up	NA	NA	NA
54	Moa03g12740.1	NA	NA	NA	NA	NA	NA	-1.08253	9.98E-05	Down	NA	NA	NA
**55**	**Moa08g11630.1**	**-2.56811**	**1.05E-09**	**Down**	**-3.43311**	**6.41E-27**	**Down**	**-3.50683**	**9.72E-14**	**Down**	**-2.66325**	**3.46E-22**	**Down**
56	Moa08g04620.1	NA	NA	NA	NA	NA	NA	NA	NA	NA	-1.02744	0.03425	Down
57	Moa13g03000.1	NA	NA	NA	NA	NA	NA	1.208797	4.77E-05	Up	NA	NA	NA
58	Moa02g09190.1	NA	NA	NA	NA	NA	NA	-2.31215	1.54E-09	Down	-1.73117	3.02E-07	Down
59	Moa08g09220.1	NA	NA	NA	NA	NA	NA	-1.36173	4.00E-09	Down	NA	NA	NA
60	Moa03g00620.1	NA	NA	NA	NA	NA	NA	NA	NA	NA	1.106074	4.34E-05	Up
61	Moa12g01550.1	NA	NA	NA	NA	NA	NA	-1.07953	0.000812	Down	NA	NA	NA
62	Moa02g06360.1	1.45021	3.59E-05	Up	NA	NA	NA	NA	NA	NA	NA	NA	NA
63	Moa10g13380.1	NA	NA	NA	NA	NA	NA	-1.01566	0.000427	Down	NA	NA	NA
64	Moa09g13910.1	NA	NA	NA	NA	NA	NA	-1.07831	0.000114	Down	NA	NA	NA
65	Moa03g14310.1	NA	NA	NA	NA	NA	NA	-1.11547	1.34E-05	Down	NA	NA	NA
66	Moa06g03210.1	NA	NA	NA	NA	NA	NA	-1.28063	0.000422	Down	-1.10221	2.42E-06	Down

67	Moa07g00940.1	NA	NA	NA	NA	NA	NA	NA	NA	NA	NA	NA	NA
68	Moa10g03540.1	NA	NA	NA	NA	NA	NA	NA	NA	NA	NA	NA	NA
**69**	**Moa11g14660.1**	**1.148802**	**0.001408**	**Up**	**1.121632**	**0.000189**	**Up**	**1.567256**	**2.18E-07**	**Up**	**1.250805**	**4.65E-07**	**Up**
70	Moa06g18220.1	NA	NA	NA	NA	NA	NA	-1.78212	0.002473	Down	NA	NA	NA
71	Moa02g10410.1	NA	NA	NA	NA	NA	NA	-1.06059	0.00342	Down	NA	NA	NA
72	pgp009	NA	NA	NA	NA	NA	NA	NA	NA	NA	3.154325	0.004001	Up
73	pgp014	NA	NA	NA	NA	NA	NA	NA	NA	NA	2.13722	0.01401	Up
74	Moa08g12220.1	NA	NA	NA	NA	NA	NA	-1.69639	0.005151	Down	NA	NA	NA
75	Moa06g09830.1	1.609079	0.000397	Up	NA	NA	NA	1.089277	0.036102	Up	1.119417	1.58E-06	Up
76	Moa04g00100.1	1.221094	9.18E-05	Up	NA	NA	NA	NA	NA	NA	NA	NA	NA
**77**	**Moa14g04910.1**	**1.116631**	**0.002834**	**Up**	**2.037206**	**4.91E-26**	**Up**	**1.407607**	**0.000609**	**Up**	**2.45481**	**2.42E-53**	**Up**
78	Moa07g17460.1	NA	NA	NA	NA	NA	NA	NA	NA	NA	2.221026	1.26E-05	Up
**79**	**Moa06g00960.1**	**-2.37763**	**0.012707**	**Down**	**-3.18256**	**1.69E-07**	**Down**	**-2.50174**	**0.007021**	**Down**	**-3.19043**	**3.61E-07**	**Down**
80	pgp033	NA	NA	NA	NA	NA	NA	NA	NA	NA	3.873737	0.002074	Up
81	**Moa11g02720.1**	**NA**	**NA**	**NA**	**NA**	**NA**	**NA**	**1.15005**	**0.015409**	**Up**	**NA**	**NA**	**NA**
82	Moa02g03620.1	NA	NA	NA	-1.1516	6.97E-09	Down	-1.70151	3.19E-14	Down	NA	NA	NA
83	pgp064	NA	NA	NA	NA	NA	NA	NA	NA	NA	5.893762	6.81E-18	Up
84	pgp065	NA	NA	NA	NA	NA	NA	NA	NA	NA	5.325621	3.62E-16	Up
85	pgp038	NA	NA	NA	NA	NA	NA	NA	NA	NA	4.202467	0.00823	Up
86	pgp068	NA	NA	NA	NA	NA	NA	NA	NA	NA	5.945551	4.16E-05	Up
87	pgp067	NA	NA	NA	7.581964	0.001138	Up	NA	NA	NA	9.224884	4.86E-13	Up
88	Moa08g04120.1	NA	NA	NA	NA	NA	NA	-1.53808	0.000361	Down	NA	NA	NA
89	pgp056	NA	NA	NA	NA	NA	NA	NA	NA	NA	5.083632	0.000106	Up
90	Moa03g14180.1	NA	NA	NA	NA	NA	NA	-1.00489	0.004345	Down	NA	NA	NA
91	Moa07g02560.1	-1.08285	0.001775	Down	-1.27684	0.00067	Down	-1.35103	0.000109	Down	NA	NA	NA
92	pgp074	NA	NA	NA	NA	NA	NA	NA	NA	NA	7.535705	6.57E-08	Up
93	pgp031	NA	NA	NA	NA	NA	NA	NA	NA	NA	7.396706	2.14E-07	Up
94	Moa03g00150.1	NA	NA	NA	NA	NA	NA	NA	NA	NA	4.43304	3.76E-12	Up
95	pgp062	NA	NA	NA	NA	NA	NA	NA	NA	NA	4.778464	0.003739	Up
96	Moa03g00240.1	NA	NA	NA	NA	NA	NA	NA	NA	NA	6.290299	4.88E-05	Up
97	Moa12g12370.1	NA	NA	NA	NA	NA	NA	NA	NA	NA	NA	NA	NA
98	Moa05g19090.1	-1.09823	1.06E-07	Down	NA	NA	NA	-1.66194	4.05E-12	Down	-1.22964	1.65E-18	Down
99	Moa05g09440.1	NA	NA	NA	NA	NA	NA	-1.20183	2.30E-05	Down	NA	NA	NA
100	Moa07g03450.1	NA	NA	NA	NA	NA	NA	NA	NA	NA	1.278016	1.08E-18	Up
**101**	**Moa03g06890.1**	**-1.18481**	**1.71E-06**	**Down**	**-1.0435**	**6.36E-05**	**Down**	**-1.93219**	**8.22E-10**	**Down**	**-1.50424**	**2.70E-11**	**Down**
102	Moa06g08770.1	-1.01568	6.58E-07	Down	NA	NA	NA	NA	NA	NA	-1.05173	4.15E-12	Down
103	Moa07g15510.1	-1.37301	0.014471	Down	NA	NA	NA	NA	NA	NA	NA	NA	NA
104	Moa08g06300.1	NA	NA	NA	NA	NA	NA	-1.19528	0.015958	Down	NA	NA	NA
105	Moa12g01220.1	-1.50943	0.000305	Down	NA	NA	NA	-1.51428	0.000226	Down	NA	NA	NA
106	pgp053	NA	NA	NA	NA	NA	NA	NA	NA	NA	6.985154	4.77E-07	Up

Note: highlighted red are genes for VIGS experiments. Blod fonts on behalf of genes expression were all changed in E1-10_vs_H32-10, E1-12_vs_H32-12, E2-10_vs_H32-10 and E2-12_vs_H32-12

Table S6. Photosynthetic and chloroplast related genes in different periods.

Number	gene_ID	E1-12_vs_E1-10			E2-12_vs_E2-10			H32-12_vs_H32-10		
		log2(FC)	FDR	Change	log2(FC)	FDR	Change	log2(FC)	FDR	Change
1	Moa12g06150.1	NA	NA	NA	NA	NA	NA	1.23641	5.80E-05	Up
2	Moa09g05340.1	-1.43425	8.89E-08	Down	NA	NA	NA	-1.38672	8.96E-06	Down
3	Moa07g03660.1	NA	NA	NA	NA	NA	NA	-1.22456	6.18E-10	Down
4	pgp057	NA	NA	NA	2.483301	0.007261	Up	NA	NA	NA
5	Moa02g12160.1	-1.14711	6.22E-20	Down	NA	NA	NA	-1.26763	1.47E-21	Down
6	Moa14g02910.1	NA	NA	NA	1.129779	1.19E-05	Up	NA	NA	NA
7	Moa05g05680.1	NA	NA	NA	NA	NA	NA	NA	NA	NA
8	pgp052	NA	NA	NA	4.350406	0.027778	Up	NA	NA	NA
9	Moa14g07620.1	-1.22297	2.68E-20	Down	NA	NA	NA	-1.027	1.48E-12	Down
10	Moa06g15270.1	1.160893	0.022194	Up	NA	NA	NA	NA	NA	NA
11	**Moa02g03470.1**	**1.401298**	**0.019354**	**Up**	**NA**	**NA**	**NA**	**NA**	**NA**	**NA**
12	Moa03g09030.1	NA	NA	NA	1.071778	1.23E-06	Up	NA	NA	NA
13	Moa04g01430.1	1.339937	0.021706	Up	NA	NA	NA	NA	NA	NA
14	Moa14g06540.1	NA	NA	NA	NA	NA	NA	1.560848	0.005866	Up
15	Moa06g10650.1	NA	NA	NA	1.429887	1.59E-12	Up	NA	NA	NA
16	**Moa09g07210.1**	**1.930438**	**3.54E-16**	**Up**	**2.702334**	**1.90E-23**	**Up**	**1.823202**	**6.29E-19**	**Up**
17	Moa12g07270.1	NA	NA	NA	NA	NA	NA	2.265184	2.96E-42	Up
18	Moa07g12550.1	NA	NA	NA	1.155213	1.23E-07	Up	NA	NA	NA
19	**Moa03g04110.1**	**1.657126**	**1.72E-22**	**Up**	**1.345726**	**1.74E-08**	**Up**	**1.11851**	**1.48E-12**	**Up**
20	Moa02g13070.1	NA	NA	NA	1.153976	8.07E-05	Up	NA	NA	NA

21	**Moa02g05330.1**	**-1.27421**	**1.22E-26**	**Down**	**-1.07507**	**1.24E-09**	**Down**	**-1.38193**	**2.48E-10**	**Down**
22	**Moa07g15800.1**	**1.706564**	**1.23E-12**	**Up**	**2.33772**	**4.74E-15**	**Up**	**1.638039**	**2.81E-10**	**Up**
23	Moa11g02010.1	1.165277	1.81E-14	Up	1.123159	1.84E-09	Up	NA	NA	NA
24	Moa08g02920.1	NA	NA	NA	1.298491	0.000185	Up	NA	NA	NA
25	Moa04g11340.1	NA	NA	NA	NA	NA	NA	1.267454	1.69E-08	Up
26	Moa05g01050.1	NA	NA	NA	NA	NA	NA	-1.09692	0.000147	Down
27	**Moa11g06970.1**	**-1.23754**	**1.86E-12**	**Down**	**-1.81591**	**1.54E-14**	**Down**	**-1.60296**	**1.05E-13**	**Down**
28	**Moa12g01710.1**	**1.257711**	**1.10E-07**	**Up**	**1.400269**	**7.06E-12**	**Up**	**1.131225**	**1.47E-05**	**Up**
29	**Moa14g05970.1**	**1.237371**	**0.000544**	**Up**	**NA**	**NA**	**NA**	**NA**	**NA**	**NA**
30	**Moa07g00770.1**	**1.05827**	**4.64E-09**	**Up**	**1.416937**	**1.64E-09**	**Up**	**1.016971**	**1.40E-06**	**Up**
31	Moa14g11510.1	-1.09483	2.16E-06	Down	NA	NA	NA	-1.05395	8.69E-07	Down
32	Moa02g12570.1	1.05193	0.026694	Up	NA	NA	NA	NA	NA	NA
33	**Moa06g17510.1**	**3.04018**	**9.56E-87**	**Up**	**1.00361**	**1.80E-05**	**Up**	**1.01741**	**3.02E-07**	**Up**
34	Moa02g05740.1	NA	NA	NA	1.104835	1.29E-05	Up	NA	NA	NA
35	Moa07g02640.1	-1.90611	0.022378	Down	NA	NA	NA	NA	NA	NA
36	Moa02g05110.1	NA	NA	NA	1.45031	2.09E-06	Up	NA	NA	NA
37	Moa14g06450.1	NA	NA	NA	1.455857	0.042216	Up	NA	NA	NA
38	Moa07g14230.1	NA	NA	NA	NA	NA	NA	NA	NA	NA
39	Moa10g06210.1	NA	NA	NA	1.529494	0.03462	Up	NA	NA	NA
40	Moa03g12740.1	1.088533	3.87E-12	Up	1.084533	1.83E-06	Up	NA	NA	NA
41	Moa08g11630.1	NA	NA	NA	1.591167	0.032759	Up	NA	NA	NA
42	**Moa12g06840.1**	**1.220278**	**5.11E-18**	**Up**	**2.203917**	**1.66E-19**	**Up**	**1.337919**	**3.49E-16**	**Up**
43	Moa02g06360.1	NA	NA	NA	1.195837	0.00049	Up	NA	NA	NA
44	Moa07g00940.1	NA	NA	NA	NA	NA	NA	1.254564	5.49E-06	Up
45	Moa10g03540.1	NA	NA	NA	1.193587	0.000213	Up	NA	NA	NA
46	pgp009	NA	NA	NA	3.057	0.029648	Up	NA	NA	NA
47	Moa07g06690.1	NA	NA	NA	1.181633	0.012311	Up	NA	NA	NA
48	Moa06g09830.1	NA	NA	NA	1.321537	1.96E-05	Up	1.270991	0.009277	Up
49	Moa01g09900.1	NA	NA	NA	1.221355	0.023519	Up	1.614927	0.013254	Up
50	Moa04g00100.1	NA	NA	NA	1.111832	4.41E-06	Up	NA	NA	NA
51	Moa14g04910.1	1.754325	3.98E-16	Up	1.876868	2.05E-12	Up	NA	NA	NA
52	Moa07g02760.1	NA	NA	NA	NA	NA	NA	1.282843	0.03671	Up
53	pgp033	NA	NA	NA	4.027848	0.016373	Up	NA	NA	NA
54	Moa04g11330.1	NA	NA	NA	1.139481	2.24E-08	Up	NA	NA	NA
55	**pgp064**	**NA**	**NA**	**NA**	**3.476556**	**0.00529**	**Up**	**NA**	**NA**	**NA**
56	pgp065	NA	NA	NA	4.139126	0.005956	Up	NA	NA	NA
57	pgp068	NA	NA	NA	4.629862	0.001567	Up	NA	NA	NA
58	pgp080	NA	NA	NA	1.837239	0.047435	Up	NA	NA	NA
59	pgp067	2.589081	0.018745	Up	3.387721	0.003002	Up	NA	NA	NA
60	Moa08g04120.1	NA	NA	NA	NA	NA	NA	-1.22033	0.000836	Down
61	pgp056	NA	NA	NA	5.560404	0.000637	Up	NA	NA	NA
62	**Moa04g17560.1**	**1.422016**	**1.60E-14**	**Up**	**1.924945**	**7.42E-14**	**Up**	**1.567247**	**4.09E-15**	**Up**
63	**Moa05g02340.1**	**1.371528**	**4.79E-14**	**Up**	**1.09714**	**5.67E-08**	**Up**	**1.101655**	**1.15E-07**	**Up**
64	Moa03g14180.1	NA	NA	NA	1.000988	0.002277	Up	NA	NA	NA
65	pgp074	NA	NA	NA	5.259192	0.000154	Up	NA	NA	NA
66	pgp031	NA	NA	NA	3.282137	0.001219	Up	NA	NA	NA
67	Moa03g00150.1	NA	NA	NA	2.902606	2.79E-06	Up	NA	NA	NA
68	pgp062	NA	NA	NA	5.328483	0.004597	Up	NA	NA	NA
69	Moa12g12370.1	-1.38253	2.36E-24	Down	NA	NA	NA	-1.27212	1.47E-13	Down
70	Moa07g08390.1	1.715586	0.040648	Up	NA	NA	NA	NA	NA	NA
71	Moa07g03450.1	-1.10189	2.54E-18	Down	NA	NA	NA	-1.39985	1.30E-08	Down
72	**Moa06g10020.1**	**1.424767**	**0.009749**	**Up**	**1.948148**	**0.002188**	**Up**	**2.449051**	**2.22E-05**	**Up**
73	Moa06g10910.1	1.567986	1.92E-12	Up	NA	NA	NA	1.301637	1.06E-07	Up
74	**Moa02g08320.1**	**1.889302**	**1.07E-06**	**Up**	**1.275046**	**0.000162**	**Up**	**1.970731**	**1.01E-08**	**Up**

Note: highlighted red fonts are genes for VIGS experiments. Blod fonts on behalf of genes expression were all changed in E1-12_vs_E1-10 E2-12_vs_E2-10 and H32-12_vs_H32-10

Table S7. Key Co-expression genes for *MaCLA1*、*MaTHIC*、*MaPSAAand MaPKP*.

ID-1	At gene-1	Gene name-1	ID-2	At gene-2	Gene name-2
Moa02g03470.1	AT4G15560	CLA1	Moa11g01600.1	AT5G53550	YSL3
			Moa12g06610.1	AT5G46800	BOU
			Moa14g07620.1	AT3G25690	CHUP1
Moa06g10020.1	AT2G29630	THIC	Moa03g01190.1	AT3G03630	CS26
			Moa04g08820.1	AT2G02150	EMB2794
			Moa05g13160.1	AT4G31850	PGR3
			Moa06g01970.1	AT1G05750	PDE247
			Moa09g02500.1	AT3G14900	EMB3120
			Moa11g08970.1	AT3G12580	HSP70
			Moa12g01710.1	AT3G54090	FLN1
			Moa12g11590.1	AT2G42010	PLDBETA1
Moa11g02720.1	AT5G52920	PKP2	Moa01g22490.1	AT2G44480	BGLU17
			Moa04g18790.1	AT3G25500	AFH1
			Moa05g10240.1	AT1G75780	TUB1
			Moa05g10440.1	AT1G75680	GH9B7
			Moa05g12790.1	AT5G25190	ESE3
			Moa07g10850.1	AT2G14960	GH3.1
			Moa07g13520.1	AT4G34200	EDA9

Table S8. Predicted protein-protein interaction network (PPI) for MaCLA1, MaTHIC and MaPKP2.

gene	Gene ID	Function	Homologous Gene ID	Identity %	At Name
MaCLA1	Moa14g00210.1	Hypothetical protein; Uncharacterized protein	AT5G13420.1	83.738	TRA2
	Moa04g08490.1	Solanesyl diphosphate synthase 3; Chloroplastic/mitochondrial	AT2G34630.2	65.721	GPS1
	Moa08g06810.1	Ribulose-phosphate 3-epimerase; Chloroplastic	AT5G61410.2	84.286	RPE
	Moa12g12490.1	Hypothetical protein; Uncharacterized protein	AT1G12230.1	71.498	/
	Moa08g10350.1	Fructose-bisphosphate aldolase	AT1G18270.1	72.568	/
	Moa08g04980.1	1-deoxy-D-xylulose 5-phosphate reductoisomerase; Chloroplastic	AT5G62790.1	83.193	DXR
	Moa05g08200.1	Putative 1-deoxy-D-xylulose-5-phosphate synthase; Chloroplastic	AT5G11380.1	69.156	/
	Moa02g03470.1	Putative 1-deoxy-D-xylulose-5-phosphate synthase; Chloroplastic	AT4G15560.1	87.593	CLA1
	Moa10g01110.1	Ribulose-phosphate 3-epimerase, cytoplasmic isoform; Belongs to the ribulose-phosphate 3-epimerase family	AT3G01850.2	80.543	/
	Moa13g07150.1	Putative 1-deoxy-D-xylulose-5-phosphate synthase; Chloroplastic	AT4G15560.1	74.119	CLA1
MaTHIC	Moa01g06610.1	Thiamine thiazole synthase 2; Involved in biosynthesis of the thiamine precursor thiazole.	AT5G54770.1	79.775	THI1
	Moa06g18180.1	Phosphoribosylamine--glycine ligase; Chloroplastic	AT1G09830.1	74.423	PUR2
	Moa04g00990.1	Phosphoribosylformylglycinamidine cyclo-ligase; Chloroplastic/mitochondrial	AT3G55010.2	76.923	PUR5
	Moa02g14620.1	Biotin synthase	AT2G43360.1	81.963	BIO2
	Moa02g01620.1	Hypothetical protein; Uncharacterized protein	AT3G16990.1	56.757	/
	Moa02g11310.1	Hydroxyethylthiazole kinase	AT3G24030.1	67.029	/
	Moa07g16490.1	Thiamine biosynthesis bifunctional protein ThiED	AT1G22940.1	74.451	TH1
	Moa06g15020.1	Phosphoribosylaminoimidazole carboxylase; Chloroplastic	AT2G37690.1	77.72	/
	Moa06g10020.1	Phosphomethylpyrimidine synthase; Chloroplastic	AT2G29630.3	86.574	THIC
MaPKP2	Moa12g12680.1	L-lactate dehydrogenase A; Belongs to the LDH/MDH superfamily	AT4G17260.1	79.603	/
	Moa11g04860.1	Hypothetical protein; Uncharacterized protein	AT2G36530.1	88.315	LOS2
	Moa08g10350.1	Fructose-bisphosphate aldolase	AT1G18270.1	72.568	/
	Moa03g10880.1	Hypothetical protein; Uncharacterized protein	AT2G36530.1	88.739	LOS2
	Moa11g02720.1	Pyruvate kinase isozyme g; Belongs to the pyruvate kinase family	AT5G52920.1	84.974	PKP2
	Moa06g10230.1	Cytosolic enolase 3	AT2G29560.1	85.593	ENOC
	Moa05g11870.1	Glucose-6-phosphate isomerase, cytosolic 1; Belongs to the GPI family	AT5G42740.1	88.288	/
	Moa09g09160.1	Pyruvate dehydrogenase e1 component subunit alpha; The pyruvate dehydrogenase complex catalyzes the overall conversion of pyruvate to acetyl-CoA and CO(2)	AT1G01090.1	86.398	PDH-E1_ALPHA
	Moa05g06570.1	Glucose-6-phosphate isomerase; Belongs to the GPI family	AT4G24620.1	80.32	PGI1

Table S9. List of primers used in experiments.

Primer Name	Sequence (5'—3')	PCR Length (bp)	Enzyme	Use
MaPDS-F	GTGAGTAAGGTTACCAACTTGTTTGGAGAGCTTGG	330	EcoRI	Vectors construstion
MaPDS-R	TGGAGGCCTTCTAGACTTTGACATGGCAATAAACAC			
MaCLA1-F	GTGAGTAAGGTTACCTCGTTCCCTGGCCATATCACC	311		
MaCLA1-R	TGGAGGCCTTCTAGAGTGAGCTCAACAACACCAAGGC			
MaPkp2-F	GTGAGTAAGGTTACCGTGGCCACACGGTCGATTCAG	355		
MaPkp2-R	TGGAGGCCTTCTAGACTCGCGTGTTCGTTGAAGGAC			
MaTHIC-F	GTGAGTAAGGTTACCGCACCACTCCCATGAGCTCAG	344		
MaTHIC-R	TGGAGGCCTTCTAGACAGTTTAGGGAGGCCAAGGCG			
QPCR-MaCLA1-F	CTTGCCCTTGATGGCCTTCTTG	180-200	/	qRT-PCR
QPCR-MaCLA1-R	GTGGTCTCCATTCAAGAGCCTC			
QPCR-MaPkp2-F	GAGATCCGTACAAGCCGAGGT			
QPCR-MaPkp2-R	TCTCTCGCGTGTTCGTTGAAG			
QPCR-MaTHIC-F	GGTTGGACCAGTTTGCGTTGT			
QPCR-MaTHIC-R	CCACGCTGCACTGCTTCTTC			
actin3-F	CACCGGTGTGATGGTTGGTATGG			
actin3-R	GGCAACACGAAGCTCATTGTAG			

*Note: Red bases are overlap with vector.
